# Running Interference? Exercise and PCB-Induced Changes in the Gut Microbiome

**DOI:** 10.1289/ehp.121-a199

**Published:** 2013-06-01

**Authors:** Carol Potera

**Affiliations:** Carol Potera, based in Montana, has written for *EHP* since 1996. She also writes for *Microbe*, *Genetic Engineering News*, and the *American Journal of Nursing*.

Trillions of diverse bacteria live in the gastrointestinal tract in a community collectively known as the gut microbiome. Changes in the makeup of the gut microbiome have been implicated in a variety of diseases, including obesity and diabetes,^1^ both of which also are affected by exercise, diet, and other lifestyle behaviors.^2^ Likewise, exposure to the industrial pollutants polychlorinated biphenyls (PCBs) has been shown to impair glucose homeostasis in mice,^3^ suggesting it could increase the risk for diabetes, and obese people have been reported to have higher body burdens of PCBs than lean people.^4^ A novel study in *EHP* looks at the interaction among the gut microbiome, exercise, and PCB exposure in mice.^5^

The study showed that short-term oral exposure to an environmentally relevant mixture of PCBs was associated with reduced abundance and diversity of gut microbes. However, mice that exercised daily before PCB dosing began showed fewer changes to the gut microbiome than sedentary mice.

The mice were about a year old, comparable to middle age for people. Half the mice exercised voluntarily on a running wheel, running an average of 10–12 km per day; the other half had locked wheels in their cages. After a five-week exercise period all mice were dosed with a mixture of PCB138, PCB153, and PCB180 for two days. These PCB congeners and the amounts given reflect those found in high concentrations in contaminated fish from the Great Lakes, according to senior author Michal Toborek, Leonard M. Miller Professor of Biochemistry and Molecular Biology at the University of Miami Miller School of Medicine. Fecal samples collected from the mice after the exercise period and again after PCB treatment were analyzed for changes in the gut microbiome.^5^

The mice that ran weighed 30% less than the sedentary animals after the five-week exercise period. In sedentary mice PCB exposure was associated with a nearly sixfold decrease in Proteobacteria, whereas mice that exercised did not experience a significant drop in Proteobacteria abundance. Compared with sedentary mice, exercised mice showed greater bacterial diversity. They had up to 24 times greater abundance of several Firmicutes species, whereas Tenericutes, Bacteroidetes, and other Firmicutes species were less abundant. The most striking difference between exercised and sedentary mice was a dramatic 360-fold reduction in Erysipelotrichaceae abundance in the former.^5^ “We didn’t expect to see so many changes in a 48-hour window,” Toborek says.

**Figure:**
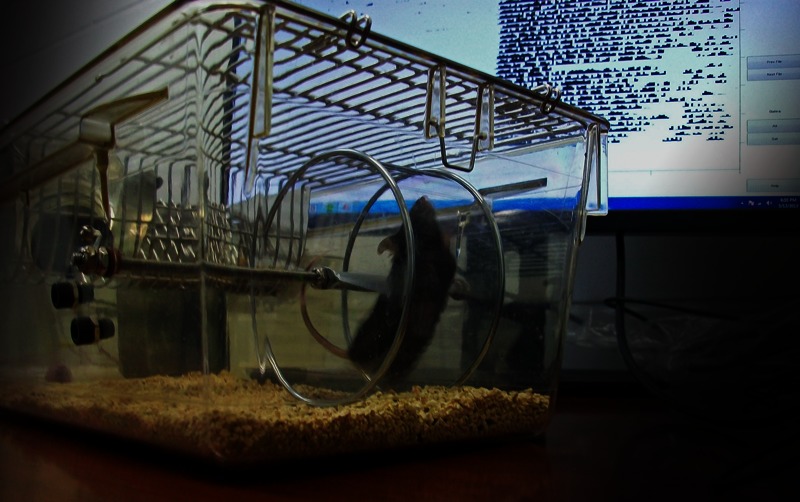
In this study, the mice’s running activity was monitored via a direct data link to a computer running Clocklab™ Analysis software. Mice that exercised before PCB exposure showed fewer changes to the gut microbiome than sedentary mice. Gretchen Wolff/University of Miami Miller School of Medicine

The physiological importance of the changes in gut bacteria remains to be determined. Other researchers have reported depletion of Firmicutes and increased levels of Proteobacteria in the guts of patients with inflammatory bowel disease^6^ and a relative abundance of Erysipelotrichaceae in patients with colorectal cancer, compared with healthy controls.^7^ Studies suggest that Bacteroidetes species are reduced and Firmicutes are elevated in obesity,^8^ and that the levels reciprocally reverse after weight loss.^9^

Toborek plans to expose mice to PCBs for longer times to examine how chronic exposure affects the gut microbial profile. Mice also will be exercised after treatment with PCBs to determine the effects of physical activity over time. Other experiments will look at the influence of the gut microbiome on the chemical fate of PCBs. Gut microbes produce enzymes that chemically alter drugs, hormones, and carcinogens. These biotransformations can either diminish or enhance the toxicity of substances such as PCBs.^10^^,^^11^

“It’s well known that diet may alter biological responses to environmental [toxicants], but the role of gut flora in toxification processes has rarely been evaluated,” says Larry W. Robertson, a University of Iowa professor of occupational and environmental health. Toborek’s results give new insights into the relationship between the gut microbiome and toxicity as well as the ways in which exercise alters the gut microbiome and toxicity. In addition to promoting diet and exercise for good health, Robertson says, perhaps we should include the role our gut flora play in wellness.
